# Effect of acute lithium administration on penile erection: involvement of nitric oxide system

**Published:** 2016-02

**Authors:** Saleh Sandoughdaran, Hamed Sadeghipour, Hamid Reza Sadeghipour

**Affiliations:** 1 *Department of Radiation Oncology, Shohada-e-Tajrish Hospital, Faculty of Medicine, Shahid Beheshti University of Medical Sciences, Tehran, Iran.*; 2 *Department of Urology, Massachusetts General Hospital, Harvard Medical School, Boston, MA, USA.*; 3 *Department of Physiology, School of Medicine, Tehran University of Medical Sciences, Tehran, Iran*

**Keywords:** *Lithium*, *Erectile dysfunction*, *Nitric oxide*

## Abstract

**Background:**

Lithium has been the treatment of choice for bipolar disorder (BD) for many years. Although erectile dysfunction is a known adverse effect of this drug, the mechanism of action by which lithium affects erectile function is still unknown.

**Objective:**

The aim was to investigate the possible involvement of nitric oxide (NO) in modulatory effect of lithium on penile erection (PE). We further evaluated the possible role of Sildenafil in treatment of lithium-induced erectile dysfunction.

**Materials and Methods:**

Erectile function was determined using rat model of apomorphine-induced erections. For evaluating the effect of lithium on penile erection, rats received intraperitoneal injection of graded doses of lithium chloride 30 mins before subcutaneous injection of apomorphine. To determine the possible role of NO pathway, sub-effective dose of N (G)-nitro-L-arginine methyl ester (L-NAME), a nitric oxide synthase (NOS) inhibitor, was administered 15 min before administration of sub-effective dose of lithium chloride. In other separate experimental groups, sub- effective dose of the nitric oxide precursor, L-arginine, or Sildenafil was injected into the animals 15 min before administration of a potent dose of lithium. 30 min after administration of lithium chloride, animals were assessed in apomorphine test. Serum lithium levels were measured 30 min after administration of effective dose of lithium.

**Results:**

Lithium at 50 and 100 mg/kg significantly decreased number of PE (p<0.001), whereas at lower doses (5, 10 and 30 mg/kg) had no effect on apomorphine induced PE. The serum Li+ level of rats receiving 50 mg/kg lithium was 1±0.15 mmol/L which is in therapeutic range of lithium. The inhibitory effect of Lithium was blocked by administration of sub-effective dose of nitric oxide precursor L-arginine (100 mg/kg) (p<0.001) and sildenafil (3.5 mg/kg) (p<0.001) whereas pretreatment with a low and sub-effective dose of L-NAME (10mg/kg) potentiated sub-effective dose of lithium, (p<0.001).

**Conclusion:**

These results suggest acute treatments with lithium cause erectile dysfunction in an in-vivo rat model. Furthermore it seems that the NO pathway might play role in erectile dysfunction associated with lithium treatment. Findings also suggest that Sildenafil may be effective in treatment of lithium-associated erectile dysfunction.

## Introduction

Although it has been 60 years since Cade first described patients who responded to anti-manic lithium treatment, international guidelines still consider lithium as a key treatment for management of bipolar disorder and recommend it in monotherapy as first-line long-term treatment (1-3). Controlled trials provide support for long-term lithium effectiveness, including for reducing risk of recurrences of bipolar depression (4-6). Moreover, lithium is most effective treatment for rapid-cycling bipolar disorders, in which depression is major component (7). However, as with many other drugs, lithium has unwanted side effects. Erectile dysfunction (ED) is one of reported lithium side effects (8-12). Ghadirian *et al* reported that a combination of lithium and other psychotherapeutic drugs, especially benzodiazepines, increased the frequency of sexual dysfunction up to half (9). In another study, Aizenberg *et al* showed that 14% of bipolar patients receiving lithium as sole medical treatment, reported having difficulties in achieving and maintaining erections (8). However, the mechanism of action by which lithium affects sexual function is still unknown.

Some studies have reported that lithium exerts its therapeutic effects through inhibition of specific targets in signaling systems, which include inositol monophosphatase, glycogen synthase kinase-3 or β-arrestin-2/Akt complex (13-16). Some authors have suggested that Nitric Oxide (NO) may be involved in some of lithium-induced responses in brain or other tissues (17-21). NO is a messenger molecule that mediates several important physiological functions (22). NO pathway is of critical importance in physiologic induction and maintenance of erections (23, 24). It has been shown in an in vitro model that lithium administration decreases the relaxation of rat corpus cavernosum and this effect is reversed by co-administration of precursor of NO, L-arginine, and is potentiated by nitric oxide synthase (NOS)inhibitor N(G)-nitro-L-arginine methyl ester (L-NAME), indicating that NO could be involved in this effect of lithium (25-27). However, exact role of L-arginine/ NO pathway in therapeutic effects of lithium is yet unidentified.

In the present study, we examined the effect of acute lithium administration on penile erection induced by subcutaneous injection of apomorphine in rats. Regarding the functional interactions of lithium with NO signaling pathway, we further investigated the possible involvement of NO in modulatory effect of lithium on penile erection using NO precursor L-arginine and NOS inhibitor L-NAME. We also evaluated the possible role of Sildenafil (phosphodiesterase 5 [PDE5] inhibitor) in treatment of lithium-induced erectile dysfunction.

## Materials and methods

This experimental study was performed between 2008 and 2014 at Tehran University of Medical Sciences. Experiments were conducted in accordance with the recommendations of the ethics committee of Tehran University of Medical Sciences.

Subjects were male Sprague-Dawley rats weighing 250-350 gr. The rats were kept 4/cage at room temperature (22-24^o^C) and with a 12 hour light/dark cycle (07.00 AM-19.00 PM). The animals had free access to food and water except during the experiments. Each rat was used only once, and each experimental group consisted of at least six animals. All experiments were performed between 10:00 AM and 15:00 PM. Animals were allowed to adapt for 30 min before first drug injection. 

Rats were placed individually into a transparent Plexiglas cage (20×20×40 cm) immediately after drug injection. Mirror was placed under observation cages to facilitate observation of animals. Rat penile erection was considered to occur when following behaviors were presented: repeated pelvic thrusts immediately followed by an upright position and an emerging, engorged penis, followed by genital grooming (28, 29). Not all behaviors were necessary to be considered a penile erectile event; only an emerging engorged penis was needed. Numbers of penile erections were counted by direct observation for 30 min after apomorphine injection.

The following drugs were used: Lithium chloride, L-arginine, L-NAME (Sigma, St Louis, MO, USA), Sildenafil (Vordin, India) and Apomorphine hydrochloride (Sigma, St Louis, MO, USA). All drugs were dissolved in saline except apomorphine hydrochloride which was dissolved in 1 mg of ascorbic acid/1 ml saline. Each rat received 0.08 mg/kg apomorphine via subcutaneous (s.c.) injection at nape of neck. For evaluating the effect of lithium on penile erection, animals received acute intraperitoneal injection of saline or graded doses of lithium chloride (5, 10, 30, 50 and 100 mg/kg) 30 min before acute subcutaneous injection of apomorphine in separate groups. In this step, both the per se sub-effective and potent doses of lithium were found for assessment in our next experiments.

For evaluating the effects of L-NAME (5, 10, 30, 50 and 100 mg/kg), L-arginine (100 mg/kg, a dose that produces no effect on sexual function), and Sildenafil (0.5, 3.5, 7 mg/kg) on penile erection, L-arginine, L-NAME and Sildenafil were administered 45 min before apomorphine test in separate groups of animals (30). In these experiments, the sub-effective doses of L-NAME and Sildenafil were found for next experiments.

For evaluating the possible role of NO pathway in the effect of lithium on PE, sub-effective dose of L-NAME was administered 15 min before administration of sub-effective dose of lithium chloride. Thirty min after lithium administration, animals were assessed in apomorphine test. In other separate experimental groups, either L-arginine (100 mg/kg) or sub-effective dose of Sildenafil was injected into animals 15 min before administration of potent dose of lithium. 30 min after administration of active dose of lithium chloride, animals were assessed in apomorphine test.

Serum lithium levels were measured 30 min after administration of effective dose of lithium (50 mg/kg). Blood was collected from heart and transferred immediately into disposable siliconized tubes and allowed to stand for 30 min. Serum was then separated by centrifugation in a micro-centrifuge (Eppendorf, Germany). Serum lithium levels were determined by an atomic absorption spectrophotometer (PerkinElmer, Norwalk, CT, USA). Comparisons between experimental and control groups were performed by one-way ANOVA followed by Newman-Keuls test when appropriate. A value of p<0.05 was considered to be significant.

**Statistical analysis**

Statistical analysis was performed using SPSS statistical software (version 18.0). Comparisons between experimental and control groups were performed by one-way ANOVA followed by Newman-Keuls test when appropriate. P<0.05 was considered as significant.

## Results

**Figure 1:**
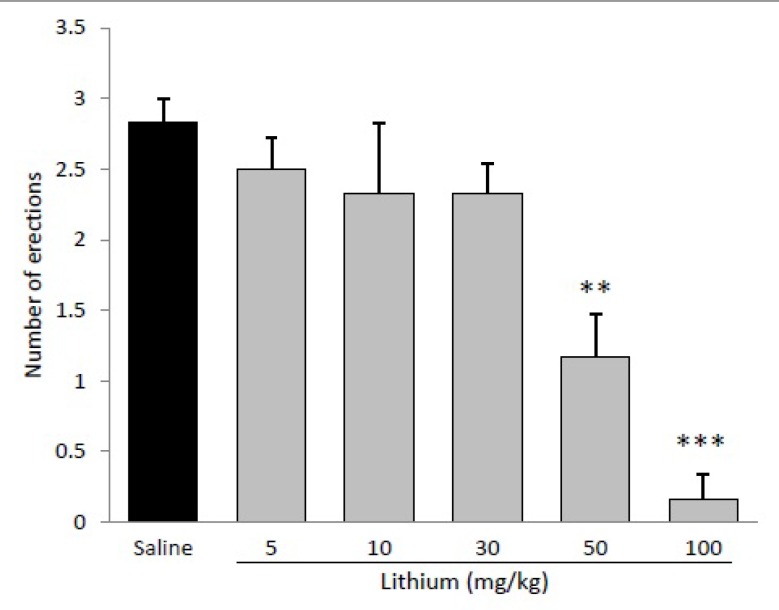
Effect of acute administration of lithium (5-100mg/kg, i.p.) on apomorphine induced penile erection in rats. Lithium was administered 30 min before apomorphine. ***p<0.001 and **p<0.01 compared with the saline-treated control. Values are expressed as mean±SEM. (n=6) and were analyzed using a one-way or two-way ANOVA followed by Newman-Keuls test

Serum level of lithium was 1±0.15 mmol /L in mice treated with 50 mg/kg lithium, and it was undetectable in control rats. Results depicted in figure 1 show that administration of lithium chloride decreased the effect of apomorphine-induced penile erection. ANOVA revealed a significant effect of lithium on penile erection (p<0.001).

**Figure 2:**
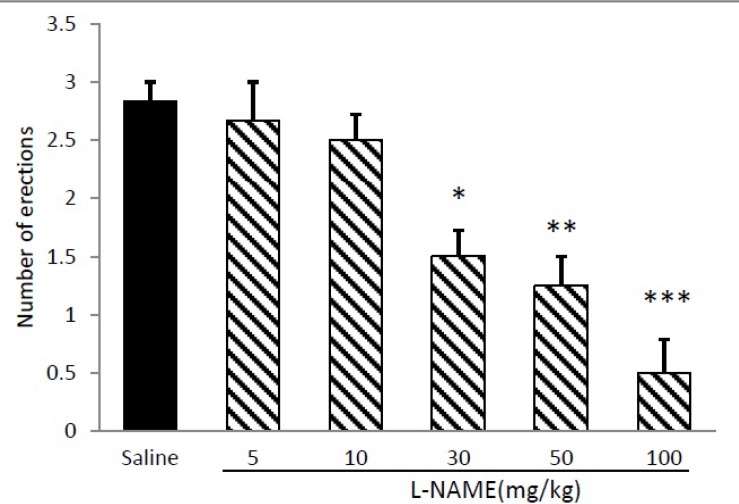
Effect of acute administration of L-NAME (5-100mg/kg, i.p.) on apomorphine induced penile erection in rats. L-NAME was administered 45 min before apomorphine. *p<0.05, **p<0.01 and ***p<0.001 compared with the saline-treated control. Values are expressed as mean±SEM. (n=6) and were analyzed using a one-way ANOVA followed by Newman-Keuls test

Lithium chloride in concentrations of 50 mg/kg (p<0.001) and 100 mg/kg (p<0.001) significantly decreased number of erections, whereas at doses of 5, 10 and 30 mg/kg didnot elicit any inhibitory effect on apomorphine-induced PE. Hence, 50 mg/kg was considered an ‘‘effective” and 30 mg/kg a ‘‘sub-effective” dose of lithium for subsequent experiments. Figure 2 illustrates the effect of different doses of L-NAME on apomorphine induced PE. L-NAME at dose of 10 mg/kg did not exert any significant effect on PE, whereas, at doses of 30 (p<0.05), 50 (p<0.01) and 100 (p<0.001) significantly decreased number of erections. 

**Figure 3:**
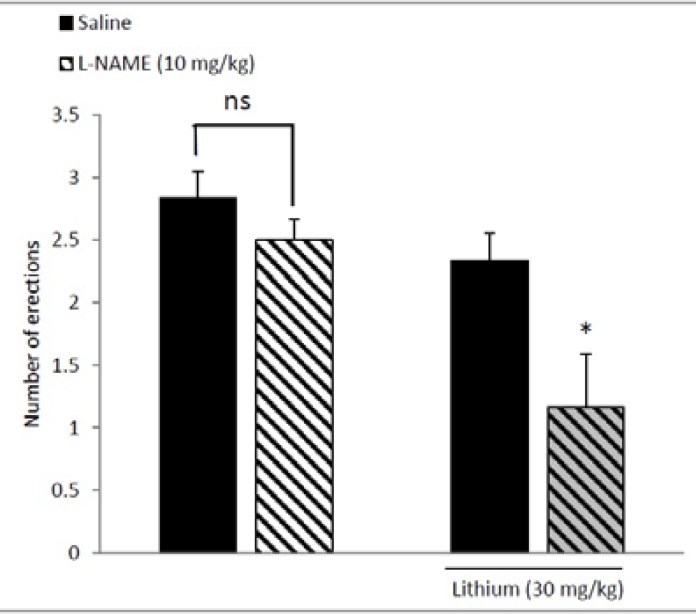
Additive effect of Lithium and L-NAME when co-administered in sub-effective doses (30 and 10 mg/kg, respectively). L-NAME or saline was administered 15 min before i.p. injection of lithium chloride and 45 min before apomorphine test. *p<0.05 compared with the saline/lithium-treated group; ns non-significant. Values are expressed as mean±SEM. Each group consisted of six animals

**Figure 4:**
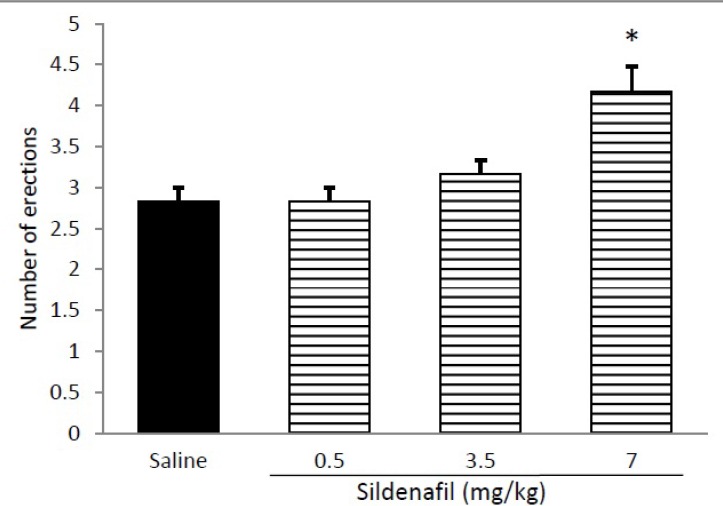
Effect of acute administration of Sildenafil (0.5-7mg/kg, i.p.) on apomorphine induced penile erection in rats. Sildenafil was administered 45 min before apomorphine. *p<0.05 compared with the saline-treated control. Values are expressed as mean±SEM (n=6) and were analyzed using a one-way ANOVA followed by Newman-Keuls test

Figure 3 shows that combination of per se non-effective doses of L-NAME (10 mg/kg) and lithium chloride (30 mg/kg) significantly (p<0.01) decreased number of erections. As shown in figure 4, Sildenafil at dose of 7 mg/kg significantly increased number of erections (p<0.05), whereas the doses of 0.05 and 3.5 mg/kg produced no significant effect on apomorphine-induced PE. 

**Figure 5:**
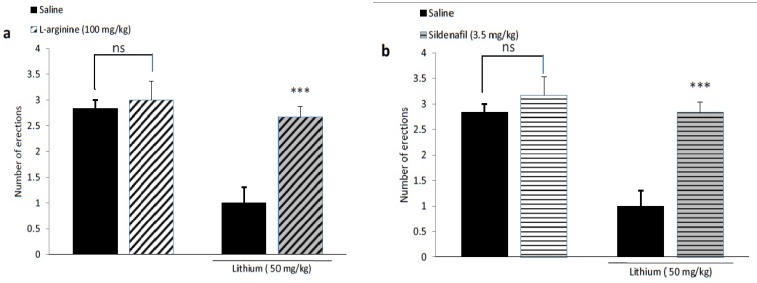
Pretreatment with a L-arginine (100 mg/kg, i.p.), or b Sildenafil (3.5 mg/kg, i.p.) 15 min before administration of lithium (50 mg/kg) abolished the inhibitory effect of lithium on penile erection. Lithium was administered 30 min before apomorphine. *** p<0.001 compared with the saline/lithium-treated group; ns non-significant. Values are expressed as mean±SEM Each group consisted of six animals

As shown in figure 5 although either L-arginine (100 mg/kg) or Sildenafil (3.5 mg/kg) individually did not affect apomorphine-induced PE, they significantly (p<0.001) reversed the inhibitory effect of 50 mg/kg lithium chloride on penile erections. 

## Discussion

The anesthetized animals have been commonly used to determine the effects of drugs on penile erection, in which the intracavernosal pressure is recorded during electro-stimulation of peripheral nerves to penis. Compared with this method conscious animal model is less invasive and can exclude potential influence of anesthesia on activity of drugs. However, this method requires a drug that does not induce erection by itself without sexual or direct penile stimulation. Apomorphine is a nonselective dopaminergic receptor agonist that activates D1-like and D2-like receptors (31). When administrated in low doses (80 µg/kg), it facilitates penile erection in rats, rabbits, and monkeys (29, 32, 33). 

Using this animal model, we observed that acute administration of lithium at 50 mg/kg and higher doses significantly decreases number of erections produced by apomorphine. Many studies have reported that lithium treatment can cause sexual dysfunction in some men and women (8-12, 34-36). Some studies reported impotence in one-third of lithium-treated male patients and even combination therapy with other psychotropic drugs such as benzodiazepines could increase the frequency of sexual dysfunction up to 50% (8-10). The most common problems in this group of patients were reduction in frequency of sexual thoughts, difficulties in achieving and maintaining erections and loss of erection during sex (8, 36). Since lithium is among most effective and frequently used medications for affective illness, such side effects, previously considered rare, show an increased frequency nowadays (12, 35). Lithium, in initial treatment of mood disorders, is generally recommended to reach plasma concentration of 0.8-1.4 mmol/L. Also, for maintenance treatment, lithium is usually given to attain plasma concentration in the range of 0.6-1.0 mmol/L (37). Consistently, our results also showed that in animals treated with 50 mg/kg lithium, the serum Li+ level was 1±0.15 mmol/L which is in therapeutic range of lithium.

NO is a key mediator of penile smooth muscle relaxation (24). NO is synthesized by the neuronal isoform of NOS in nitrergic nerves, and by endothelial isoform (eNOS) in endothelium. NOS enzymes metabolize L-arginine to L-citrulline and NO. Both isoforms are present in penis, neuronal NOS (nNOS) in nerve terminals of penile autonomic nerves and eNOS in endothelial cells covering the cavernosal smooth muscle cell (38, 39). Penile tumescence is achieved by a collaboration of these two NOS signaling pathways (40). Any modification in either of these NO signaling pathways can cause erectile dysfunction (41).

In the present study, we examined effect of simultaneous administration of lithium with NOS inhibitor L-NAME, NO precursor L-arginine and Sildenafil on apomorphine induced PE. We observed neither low concentrations of L-NAME nor low concentrations of lithium, when administered independently, significantly affected penile erection. However, when L-NAME and lithium were combined at same low doses, they exerted a significant inhibitory effect on apomorphine induced PE. Furthermore we demonstrated that effect of active dose of lithium on PE was blocked by co-administration of dose of L-arginine which per se didnot exert any significant effect on apomorphine-treated animals.

Interestingly our results show that decreasing effect of lithium on penile erection could be restored to normal level by administration of Sildenafil. Sildenafil potentiates the effect of NO pathway through inhibition of PDE5 activity. After production, NO binds to a receptor on the enzyme guanylate cyclase, resulting in increased cGMP levels, which causes smooth muscle relaxation and promotes erection (42). cGMP is finally degraded by enzyme PDE. Sildenafil is a PDE5 inhibitor, which prolongs and enhances target cell response to NO (43).

Series of studies demonstrate the actual interaction between lithium and NOS activity in different tissues (44, 45). Using an in vivo brain microdialysis method, Maruta *et al* demonstrated that NO3 and NOX levels were significantly reduced in rat amygdale between 60 and 90 min after intraperitoneally lithium administration (46). Moreover, Dehpour *et al* reported the additive effect of chronic lithium treatment when co-administered with L-NAME on withdrawal signs and physical dependence in morphine-dependent mice (47). In another study, it was reported that high doses of lithium caused a significant decrease in NOS activity of rat hippocampus (48). Also, Hashioka *et al* have shown that 24 hr pretreatment with lithium chloride could inhibit NO production by interferon-c-activated microglia in a dose-dependent manner. Recently, it has been reported that chronic lithium treatment decreases the relaxation of rat corpus cavernosum and this inhibitory effect of lithium is prevented by co-administration of a sub-effective dose of L-arginine and is potentiated by concurrent administration of a low and sub-effective dose of L-NAME (25). 

It has also been shown that NO system could be involved in the antidepressant-like effects of lithium in the mouse forced swimming test (49, 50). Taken together and according to our study, it seems reasonable that lithium elicits some of its effects on PE at least in part, via modulation of NO pathway.

## Conclusion

In conclusion, we report here that acute treatments with lithium cause erectile dysfunction in an in vivo rat model. We also found that low doses of lithium show a strong inhibitory eﬀect together with low and sub-effective dose of L-NAME on apomorphine-induced PE. Further, L-arginine, at per se sub-effective concentrations, signiﬁcantly blocked the inhibitory effect of lithium. Therefore, it seems that NO pathway might play a role in erectile dysfunction associated with lithium treatment. The findings also suggest that Sildenafil may be effective in the treatment of lithium-associated erectile dysfunction.
